# Neural stem cell-like cells derived from autologous bone mesenchymal stem cells for the treatment of patients with cerebral palsy

**DOI:** 10.1186/1479-5876-11-21

**Published:** 2013-01-26

**Authors:** Guojun Chen, Yali Wang, Zhenyu Xu, Feng Fang, Renmei Xu, Yue Wang, Xiaoli Hu, Lixing Fan, Houqi Liu

**Affiliations:** 1 Division of Pediatrics, Zhejiang General Hospital of Armed Police Forces, 16 South Lake Road, Jiaxing City, 314000, China; 2 Research Center of Developmental Biology, Second Military Medical University, 800 Xiangyin Road, Shanghai, 200433, China

**Keywords:** Cerebral palsy, Neural stem cell-like cells, Bone marrow mesenchymal stem cells, Cell therapy, Autologous transplantation

## Abstract

**Background:**

Stem cell therapy is a promising treatment for cerebral palsy, which refers to a category of brain diseases that are associated with chronic motor disability in children. Autologous MSCs may be a better cell source and have been studied for the treatment of cerebral palsy because of their functions in tissue repair and the regulation of immunological processes.

**Methods:**

To assess neural stem cell–like (NSC-like) cells derived from autologous marrow mesenchymal stem cells as a novel treatment for patients with moderate-to-severe cerebral palsy, a total of 60 cerebral palsy patients were enrolled in this open-label, non-randomised, observer-blinded controlled clinical study with a 6-months follow-up. For the transplantation group, a total of 30 cerebral palsy patients received an autologous NSC-like cells transplantation (1-2 × 10^7^ cells into the subarachnoid cavity) and rehabilitation treatments whereas 30 patients in the control group only received rehabilitation treatment.

**Results:**

We recorded the gross motor function measurement scores, language quotients, and adverse events up to 6 months post-treatment. The gross motor function measurement scores in the transplantation group were significantly higher at month 3 (the score increase was 42.6, 95% CI: 9.8–75.3, *P*=.011) and month 6 (the score increase was 58.6, 95% CI: 25.8–91.4, *P*=.001) post-treatment compared with the baseline scores. The increase in the Gross Motor Function Measurement scores in the control group was not significant. The increases in the language quotients at months 1, 3, and 6 post-treatment were not statistically significant when compared with the baseline quotients in both groups. All the 60 patients survived, and none of the patients experienced serious adverse events or complications.

**Conclusion:**

Our results indicated that NSC-like cells are safe and effective for the treatment of motor deficits related to cerebral palsy. Further randomised clinical trials are necessary to establish the efficacy of this procedure.

## Background

Childhood cerebral palsy is a non-progressive brain disease that results from various cerebral insults that can occur before birth and 1 month after birth. Patients primarily present with motor developmental delay or motor dysfunction and possible mental retardation, epilepsy, behavioural disorders, and sensory and perceptual disturbances. Hypoxic ischemic encephalopathy and premature cerebral palsy are the most common causes of cerebral palsy [1,2]. A census conducted in six cities in 1998 revealed that the prevalence of cerebral palsy in children 1 to 6 years of age was 1.92% [3]. The incidence of cerebral palsy in pre-schoolers in the United States has ranged from 3%–4% [4]. Children with cerebral palsy may impart a heavy burden on their families and society. Conventional therapies for treating cerebral palsy include physical therapy, motor function training, language training, orthomorphia, neurotomy, and intramuscular injections of botulinum toxin A. However, these methods have not improved cerebral injuries in patients with moderate-to-severe cerebral palsy [5,6].

Stem cell transplantation is a novel and promising treatment for cerebral palsy [7,8]. However, this procedure is still in the initial stages of investigation [9], and there have not been any published results from clinical trials to date [10]. Several types of stem cells are candidates for the treatment of cerebral palsy, such as human embryonic neural stem cells, olfactory ensheathing cells, umbilical mesenchymal stem cells, and bone marrow MSCs [7,8,11,12]. However, human embryonic neural stem cells and olfactory ensheathing cells are difficult to apply in clinical practice due to the potential immunological rejection of xenogenic cells, ethical arguments, a high risk of transplantation within the brain, and the difficulty of repeated transplantations [9]. Autologous MSCs may be a better cell source and have been studied for the treatment of cerebral palsy because of their functions in tissue repair and the regulation of immunological processes [13]. In addition, previous studies have demonstrated that human bone marrow MSCs exhibit neural phenotypes and can differentiate into NSC-like cells in vitro [14-16]. Moreover, intraspinal cell therapy has led to better outcomes for neurological disorders in animal-based studies [17,18] and clinical trails [19,20]. Therefore, we hypothesized that an intraspinal infusion of autologous MSCs-derived NSC-like cells may be a novel treatment for patients with moderate-to-severe cerebral palsy. In a controlled clinical study, we investigated the clinical outcomes of 30 cerebral palsy patients who underwent transplantation with NSC-like cells and who were followed up for 6 months.

## Methods

### Study design and patient enrolment

**Figure 1:**
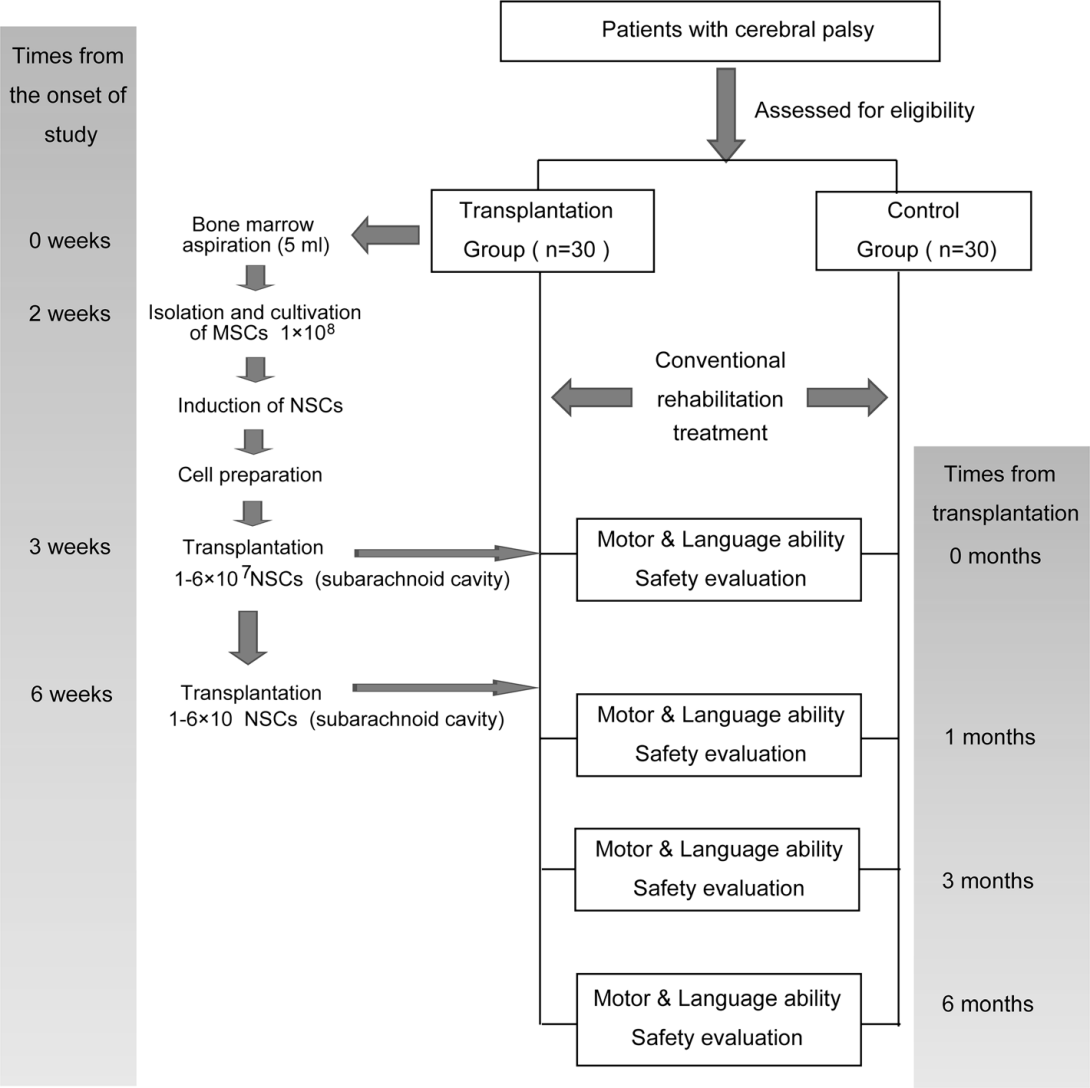
**Study protocol.** A total of 60 patients with cerebral palsy with Gross Motor Function Classification System (GMFCS) levels III-V were enrolled in this study. For the transplantation group, a total of 30 cerebral palsy patients received the autologous NSC-like cell transplantation and rehabilitation treatments whereas 30 patients in the control group only received rehabilitation treatment. The study protocol is shown. Motor ability was assessed using the Gross Motor Function Measure questionnaire-88. Language ability was measured using the Gesell questionnaire. All of the assessments were conducted by a specifically assigned person, and the assessment results were recorded in the case report. A safety evaluation was performed during the 6-month follow-up to detect instances of death, any serious clinical events, abnormal electroencephalogram results, and neuroimaging complications.

An open-label, non-randomised, observer-blinded controlled clinical study was conducted. The study was approved by the Ethics Committee and the Science Committee of the Armed Police General Hospital of Zhejiang Province, China and was registered in the China Clinical Trial Registry (ChiCTR-TRC-12002056). Informed consent was obtained from all of the patients or their parents. A total of 60 patients with cerebral palsy with Gross Motor Function Classification System (GMFCS, corresponding to GMFCS E&R 2007) levels III-V were enrolled in this study from June 2010 to June 2011. Overall, 30 patients were treated with conventional methods and stem cell transplantation and were included in the transplantation group. An additional 30 patients with cerebral palsy were only treated with conventional methods and were included in the control group. The study protocol is shown in Figure 1.

The patients were enrolled according to the following inclusion criteria [3]: (1) the presence of non-progressive, neurological disorders that appeared in infancy or early childhood; (2) the appearance of retardation including the lack of development or ability in sporting activities and the persistence of posture obstacles, which may be associated with lesions that affect feeling, perception, cognition, communication, and behaviour, epilepsy or secondary musculoskeletal problems; (3) no diagnosis of other diseases, such as metabolic or degenerative diseases, that could induce central paralysis and transient retardation in a normal person; and (4) negative serological markers for AIDS, hepatitis, and syphilis. Patients with severe anaphylactic or autoimmune diseases were excluded from the transplantation group.

### Autologous MSC cultures

Patients fasted for 4–6 hours and went without water 1 hour before the collection of autologous bone marrow specimens. The patients were injected with 5 mg/kg phenobarbital, 0.01 mg/kg atropine, and 4 mg/kg ketamine in the aseptic collecting room 30 minutes before specimen collection. In addition, 8–25 mL of bone marrow (0.6-1.0 mL/kg of body weight) was collected from the right posterior superior iliac spine according to conventional bone marrow aspiration procedures. A total of 100 U/mL heparin was added to the collecting tube as an anticoagulant. Following current manufacturing best practices, mononuclear bone marrow cells were isolated by Percoll (1.073 g/mL) centrifugation and were allowed to adhere to a flask for 72 hours in low glucose Dulbecco’s Modified Eagle’s Medium (Gibco-Invitrogen) at 5% CO_2_ and 37°C, and the media were changed every 3 days. The cells with a strong positive signal for the multipotential marker Oct 4 and Nanog were screened for further cloning and culturing (Additional file 1: Figure S1A). The cell phenotypes were assessed by flow cytometry. The results indicated that CD34 and CD45 were negative, and the positive rates of CD29 and CD44 were above 95% (Additional file 1: Figure S1B). The ability of the cells to differentiate into adipocytes and osteocytes in culture was confirmed in vitro following the criteria of the 2006 International Society of Cellular Therapy [21]. At 70%–80% confluence, the cells were detached and replated at 5 × 10^6^/175 cm^2^ in culture to process for neural differentiation and confirm negativity for endotoxin, hepatitis C virus, hepatitis B virus, HIV, syphilis, fungus, *Myco plasma* species, and *Chlamydia* before infusion. A G-banding karyotype analysis was performed to confirm the absence of chromosomal aberrations in the final cellular product. The MSCs were continuously cultured without cryopreservation and were thawed before transplantation. Supplementary methods were shown in more detail (see Additional file 1).

### Autologous NSC-like cells induction and differentiation

**Figure 2:**
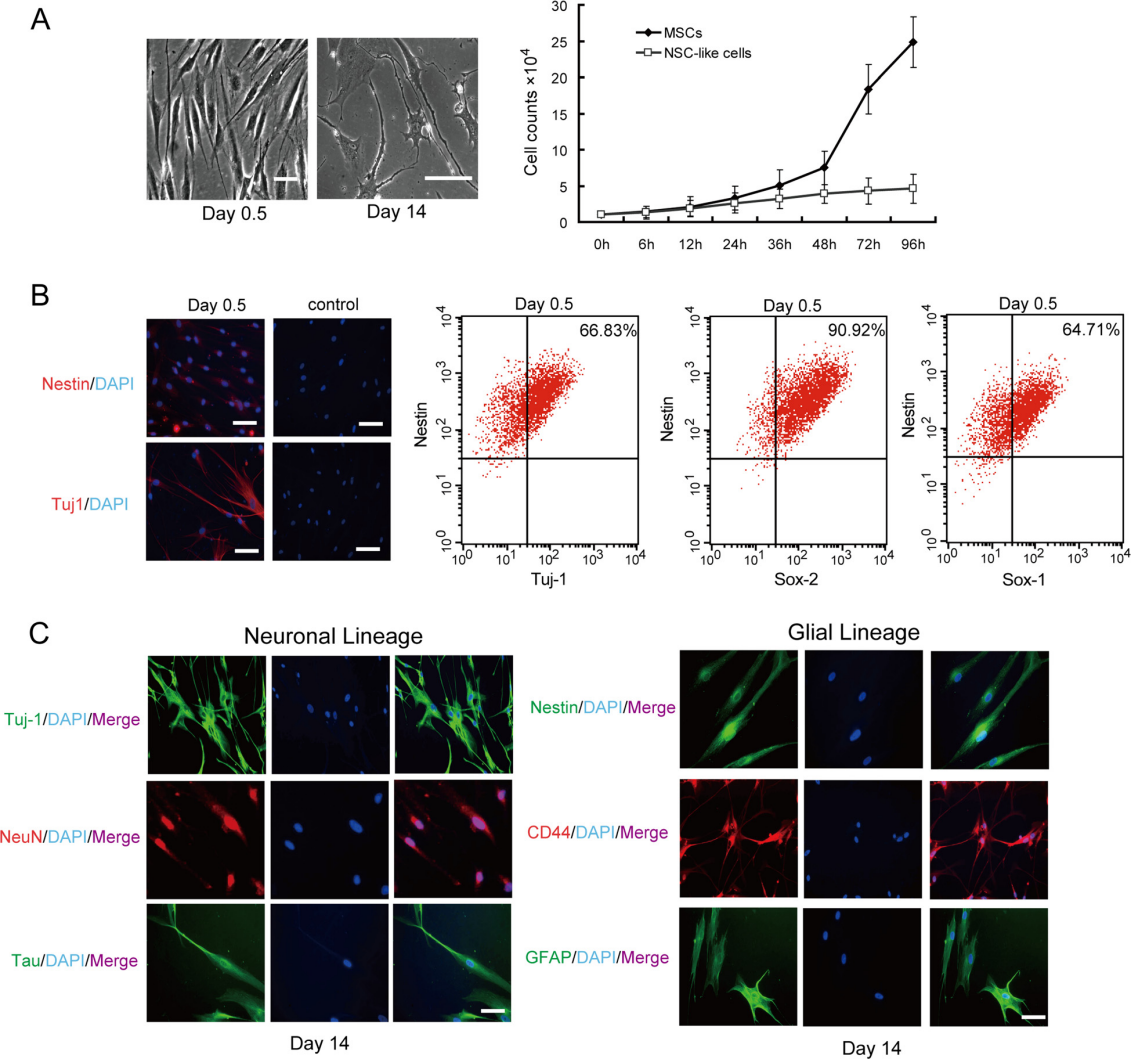
**MSCs can be induced to differentiate into NSC-like cells and exhibit multipotency by generating progenies of different neural lineages in vitro. (A)** During differentiation, MSCs develop the properties of neural stem cells (NSCs). The induced cells were observed in the bright fields after 0.5 days and after 14 days. The growth curves of the MSCs and the NSC-like cells, which were obtained by enumerating the cells at each time point under a haemocytometer, are shown (*n*=3; the calculated doubling times were 20 hours and 40 hours, respectively). **(B)** After induction, the NSC-like cells were fixed, and immunofluorescence was used to detect the expression of Nestin and Tuj-1. The cells were counterstained using DAPI. The scale bar is 10 μm. To assess the induction efficiency, the cells were co-labelled with antibodies against Nestin and Tuj-1, Sox-2 or Sox1, and the proportions of double-positive cells were 66.83%, 90.92% and 64.71%, respectively. The tests were repeated three times. **(C)** To determine whether the induced NSC-like cells could differentiate into both neurons and glia cells, MSCs were induced for 14 days under the same conditions and stained using antibodies against neuron- and glial cell-specific proteins. The scale bar is 10 μm.

At 30%-40% confluence, a combination of 20 ng/ml recombinant human FGF basic (bFGF) and 10 nM retinoic acid was added to the medium for 12 hours to transform the MSCs, and the differentiation capacity was evaluated after 14 days [14]. Growth curves were drawn to assess the proliferation ability of the MSCs and the transformed cells (Figure 2A). In total, 1 × 10^4^ of the transformed cells were collected to prepare slices for immunofluorescence staining, and 50%–70% of the cells expressed Nestin and Tuj-1 (Figure 2B). A total of 1 × 10^6^ cells were co-labelled with antibodies against Nestin (BD Pharmingen) and Tuj-1 (Abcam) , Sox2 (Abcam) or Sox1 (Santa Cruz). According to the flow cytometry analysis, 66.83%, 90.92% and 64.71% of the induced cells expressed both Nestin and Tuj-1, Sox2 and Sox1, respectively (Figure 2B). These results suggest that the MSCs differentiated into NSC-like cells. The NSC-like cells were detached, washed three times in saline, re-suspended in saline with 5% human albumin and filtered in a 100-μm nylon filter. Approximately 100 μl of this solution was used for safety and viability tests. No chromosomal aberrations or increased telomerase activity was found for the induced NSC-like cells. In addition, a high cell viability (>90%) and the negative microbiological results ensured the quality of the cells before the transplantations. To determine whether the induced NSC-like cells could differentiate into different neural lineages, MSCs were induced for 14 days under the same conditions and were stained using antibodies against Tuj-1 (Abcam), NeuN (Abcam), Tau (Abcam), Nestin (Santa Cruz), CD44 (Santa Cruz) and GFAP (Santa Cruz). The induced cells expressed both neuron- and glial cell-specific proteins (Figure 2C).

### Safety evaluation of NSC-like cells in vivo in a mouse model

The MSC-derived NSC-like cells (1.0 × 10^6^) were implanted subcutaneously into the flanks of nude mice or were transplanted intraspinally into the subarachnoid space of nude mice. No tumour formation or other adverse events were observed in mice after 3 months. The animal studies were approved by the Institutional Animal Care and Use Committee of the Second Military Medical University in Shanghai, China.

### Cell transplantation therapy

Before the lumbar puncture, the patients fasted without food and water. The patients were injected with phenobarbital sodium, atropine, and ketamine according to the dose and usage guidelines before the collection of bone marrow. When the patients fell asleep, a local anaesthetic was infiltrated under the skin and a spinal needle was inserted between the lumbar vertebrae L3/L4 or L4/L5 and pushed in until the needle traversed the dura mater and the thin arachnoid membrane into the subarachnoid space. The stylet from the spinal needle was withdrawn, and drops of cerebrospinal fluid (up to 5 ml) were collected. Then, 5 ml cell suspension, which contained 1-2 × 10^7^ of NSC-like cells, was gently injected into the subarachnoid cavity through the spinal needle. The opening pressure of the cerebrospinal fluid was measured during specimen collection using a simple column manometer. The procedure ended by withdrawing the needle while placing pressure on the puncture site. The cell transplantation was administered at intervals of 3 weeks for two cycles. In addition, the patients in the transplantation group received rehabilitation treatments at the same time.

### Observation indices

As the primary measurement, the motor and language abilities of the patients were observed before treatment and at 1, 3, and 6 months post-treatment. Motor ability was assessed using the Gross Motor Function Measure (GMFM-88 or GMFM) [22] questionnaire. An example was shown in Additional file 1. Language ability was measured using the Gesell questionnaire. The language developmental quotient (LDQ) was determined using the following formula: language developmental age/present age × 100. All of the assessments were conducted by a specifically assigned person. For the observer-blinded design of the study, the patients were randomly arranged before the tests, and the examiner was blinded to the names and the grouping of patients. The assessment results were recorded in the case reports.

The secondary measurement was patient survival and the incidence of the following adverse events: (1) fever, headache, allodynia, vomiting, infection, or other reactions; (2) clinical seizures and/or epileptic discharges on a serial electroencephalogram (EEG) at any time during hospitalization; (3) new lesions in the skull according to MRI at 6 months post-procedure; and (4) other clinically significant complications from the procedure during the long-term folow-up.

### Statistical analysis

The data were expressed as the mean ± S.E.M. The statistical significance of the patient ages, GMFM scores and language developmental quotients was detected using the one-way ANOVA analysis, LSD test and two-tailed Student’s *t*-test. The statistical significance of gender and the GMFCS levels was detected using the chi-squared test. The statistical analysis was performed using SPSS 11.0.

## Results

### Baseline characteristics of the patients

**Figure 3:**
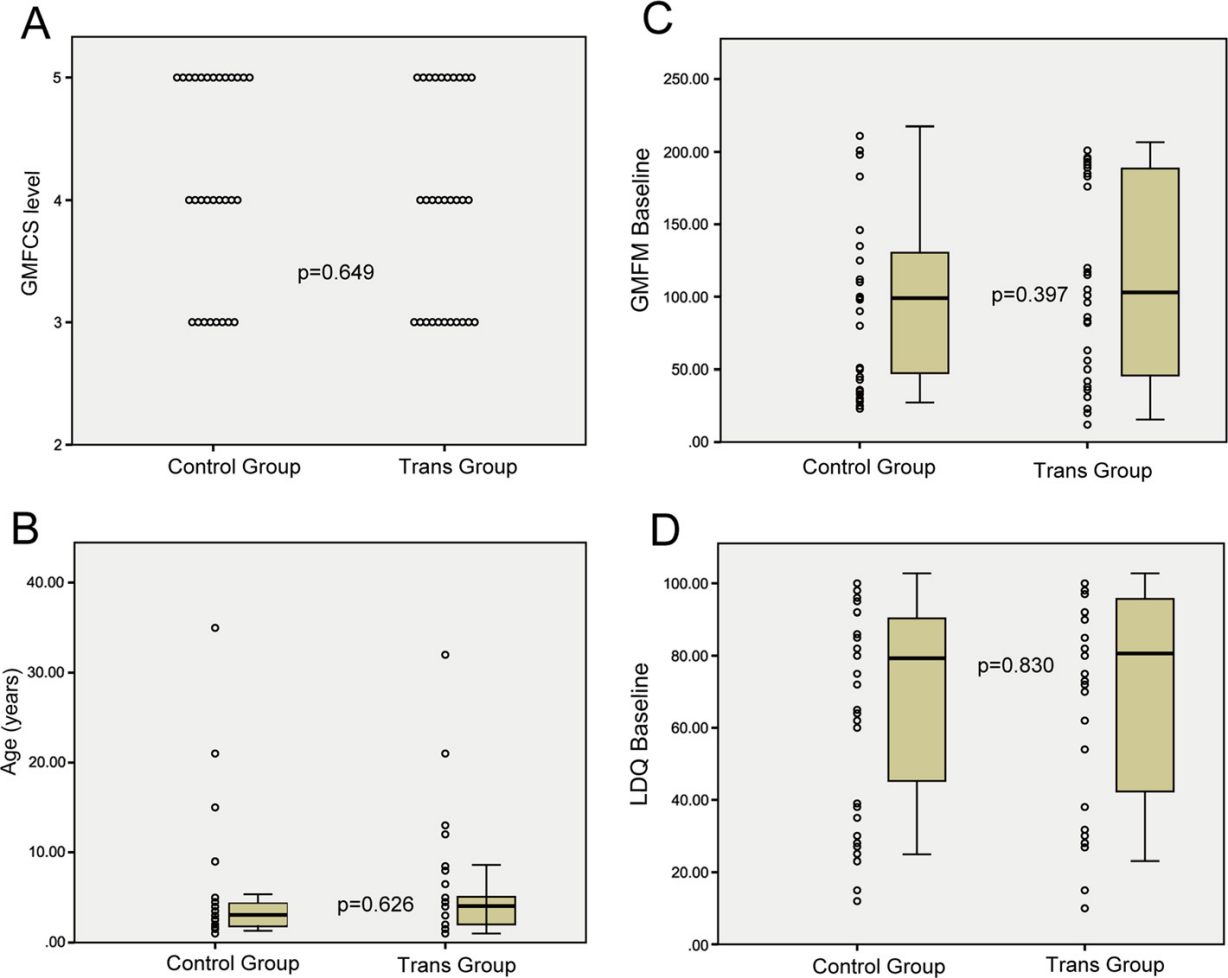
**A comparison of the baseline characteristics of the patients in the two groups. (A)** A comparison of the number of patients according to the Gross Motor Function Classification System (GMFCS) levels in the transplantation (Trans) and control groups. Statistically significant differences were detected using the chi-squared test. **(B-D)**: A comparison of the mean ages **(B)**, mean baseline Gross Motor Function Measure (GMFM) scores **(C)** and mean baseline language developmental quotients (LDQs) **(D)** of the patients in the transplantation (Trans) and control groups is shown. Statistically significant differences were detected between the groups using a Student *t*-test.

A total of 30 patients with cerebral palsy in GMFCS levels III-V were enrolled in this study and were included in the transplantation group. The group consisted of 14 males and 16 females with a mean age of 5.53 ± 1.20 years (range, 1–32 years). An additional 30 patients with cerebral palsy were treated with conventional methods and were included in the control group, which consisted of 14 males and 16 females with a mean age of 4.66 ± 1.31 years (range, 1–35 years). The baseline characteristics of the patients in the two groups at the study onset are shown in Tables 1–2. In addition, a comparison of the patient ages, LDQs and GMFM scores in the two groups are shown in Figure 3. No significant differences were found.

**Table 1:** Clinical characteristics of the 30 patients in the transplantation group

**No.**	**Gender**	**GMFCS stage**	**Age (years)**	**LDQ**	**GMFM**
1	Female	V	1	72	36
2	Female	III	3	31.7	176
3	Male	III	4	28	189
4	Male	III	4	92	185
5	Male	V	3	100	50
6	Male	IV	2	38	101
7	Male	V	4.5	15	23
8	Male	IV	4	26.8	115
9	Female	V	3	75	42
10	Female	IV	13	82	105
11	Female	V	1.5	62	38
12	Male	V	1	70	31
13	Female	V	1	75	20
14	Male	IV	2	90	63
15	Male	III	4	98	191
16	Female	IV	3	30	96
17	Male	IV	21	80	82
18	Female	III	2	98	120
19	Male	IV	5	85	86
20	Female	III	8	10	115
21	Female	III	4	90	183
22	Female	V	5	10	23
23	Female	III	12	100	195
24	Male	IV	2	98	83
25	Male	V	1	15	12
26	Female	III	6.5	90	193
27	Female	III	4	92	196
28	Female	IV	32	73	117
29	Female	V	1	54	56
30	Male	III	8.5	97	201

The clinical characteristics of the 30 patients in the transplantation group before the cell transplantation treatment are shown. GMFCS: Gross Motor Function Classification System levels; LDQ: language developmental quotient; GMFM: Gross Motor Function Measurement scores.

**Table 2:** Clinical characteristics of the 30 patients in the control group

**No.**	**Gender**	**GMFCS stage**	**Age (years)**	**LDQ**	**GMFM**
1	Female	V	1	72	36
2	Female	III	2.5	35	183
3	Male	III	3.5	28	198
4	Male	III	4	92	211
5	Male	V	2	100	35
6	Male	V	2	38	23
7	Male	V	4.5	15	33
8	Male	IV	3.5	27	110
9	Female	V	2	75	51
10	Female	IV	2.5	82	99
11	Male	IV	1	60	80
12	Female	III	15	95	201
13	Female	III	1	62	135
14	Male	IV	5	86	110
15	Female	IV	2.5	95	98
16	Male	IV	21	98	135
17	Male	IV	35	82	90
18	Male	IV	9	80	112
19	Female	V	4	12	30
20	Female	V	1	23	43
21	Male	V	1.7	25	45
22	Female	V	1	30	50
23	Female	III	1.5	80	146
24	Female	V	3	39	25
25	Male	IV	2.5	96	99
26	Male	V	2.6	100	28
27	Female	III	2	86	100
28	Female	V	1	65	45
29	Male	III	1.5	85	125
30	Male	V	1	64	43

The clinical characteristics of the 30 patients in the control group are shown. GMFCS: Gross Motor Function Classification System levels; LDQ: language developmental quotient; GMFM: Gross Motor Function Measurement scores.

The MRI results from the skulls of patients with cerebral palsy can show a periventricular white matter injury (PWMI), and the pathological findings can include periventricular leukomalacia and diffused dysmyelination [23,24]. Prasad [25] studied 102 children 1–3 years of age with cerebral palsy and found that 47.1% had a PWMI in the skull based on MRI findings. In this study, 16 of the 30 patients (54.5%) in the transplantation group had white matter injuries according to MRI.

### Functional outcomes of motor development among the two groups

**Figure 4:**
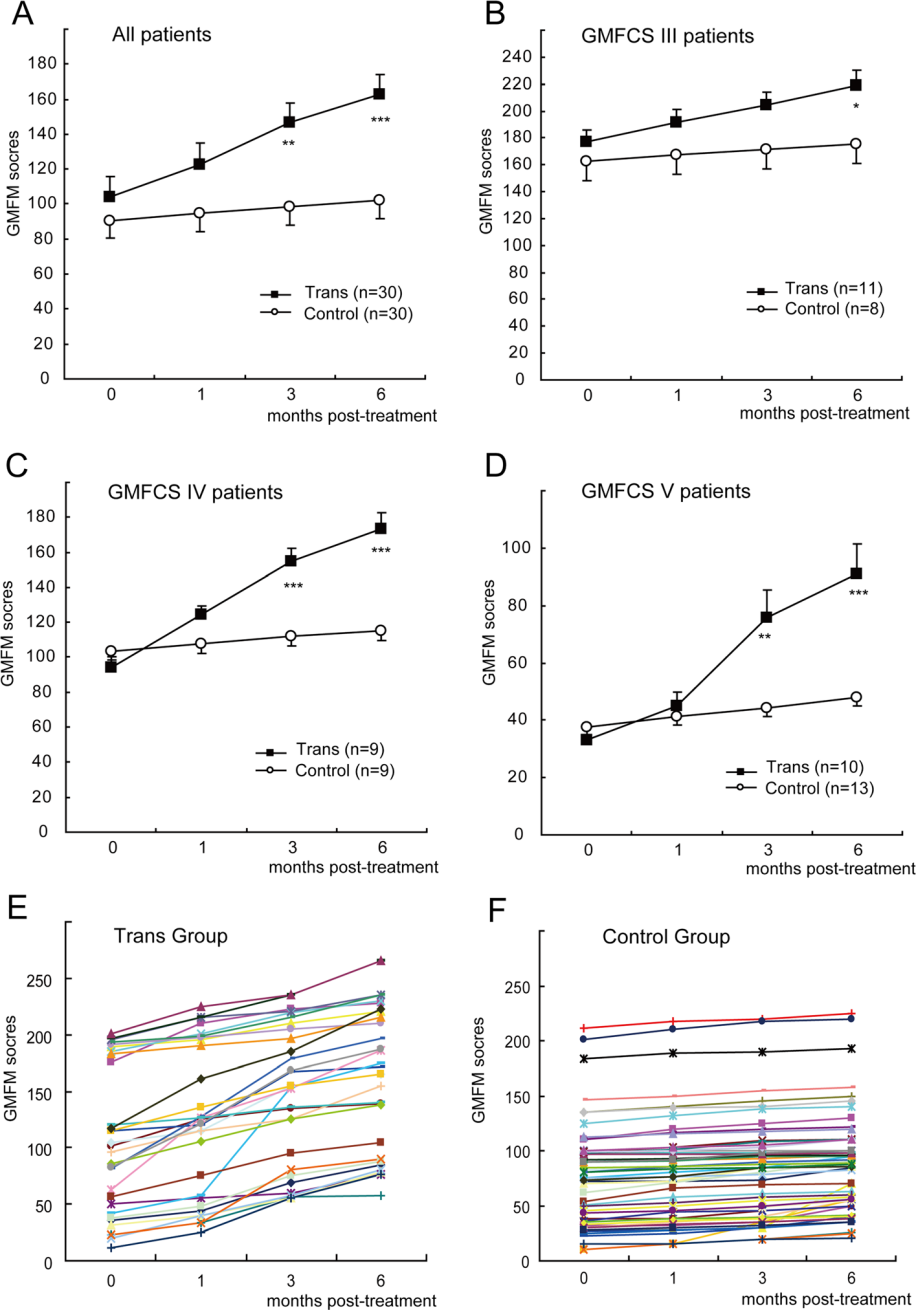
**A comparison of the kinetic Gross Motor Function Measure (GMFM) scores of the patients in the two groups. (A)** A comparison of the mean GMFM scores before treatment (0 months) and at 1, 3, and 6 months post-treatment for the patients in the transplantation (Trans) and control groups. **(B-D)** A comparison of the mean GMFM scores for the patients with GMFCS levels III **(B)**, IV**(C)** and V**(D)** in the transplantation (Trans) and control groups. The data were expressed as the mean ± S.E.M. Statistically significant differences were detected between the groups using a two-tailed Student’s *t*-test, * *P<*0.05, ** *P<*0.01, *** P<0.001. **(E-F)** Changes in the GMFM scores of the patients in the transplantation (Trans) group **(E)** and in the control group **(F)** before and after treatment. Each line indicates the kinetic scores of an individual patient.

The motor functions of the patients were observed and evaluated using the GMFM scores before treatment and at 1, 3, and 6 months post-treatment (Table 1). The GMFM scores of all of the patients in the two groups gradually increased during the 6 months post-treatment, however, there were no significant differences in the control group. In contrast, the GMFM scores in the transplantation group at months 3 and 6 post-treatment were significantly higher compared with the baseline scores (*P=*0.011 and 0.001), whereas there were no significant changes in the GMFM scores at month 1 post-treatment (*P=*0.265). The GMFM score increase from baseline to month 6 post-treatment was 58.6 (95% CI: 25.8-91.4) in the transplantation group. This finding suggests that there was a significant recovery effect in motor function after the transplantation treatment. Additional movie files were shown in more detail (see Additional file 2 and 3). In addition, we found that the GMFM scores at months 3, and 6 post-treatment were significantly higher in the transplantation group compared with those in the control group (*P=*0.003 and *P<*0.001, respectively). However, there were no differences in the GMFM scores at month 1 post-treatment between the two groups (*P=*0.089) (Figure 4A). To evaluate the impact of NSC transplantation in patients with different levels of cerebral palsy, we divided the patients according to their GMFCS levels. The results indicated that patients in the transplantation group with levels IV and V had a better recovery of motor function (Figure 4B-D).

### Functional outcomes of language development among the two groups

**Figure 5:**
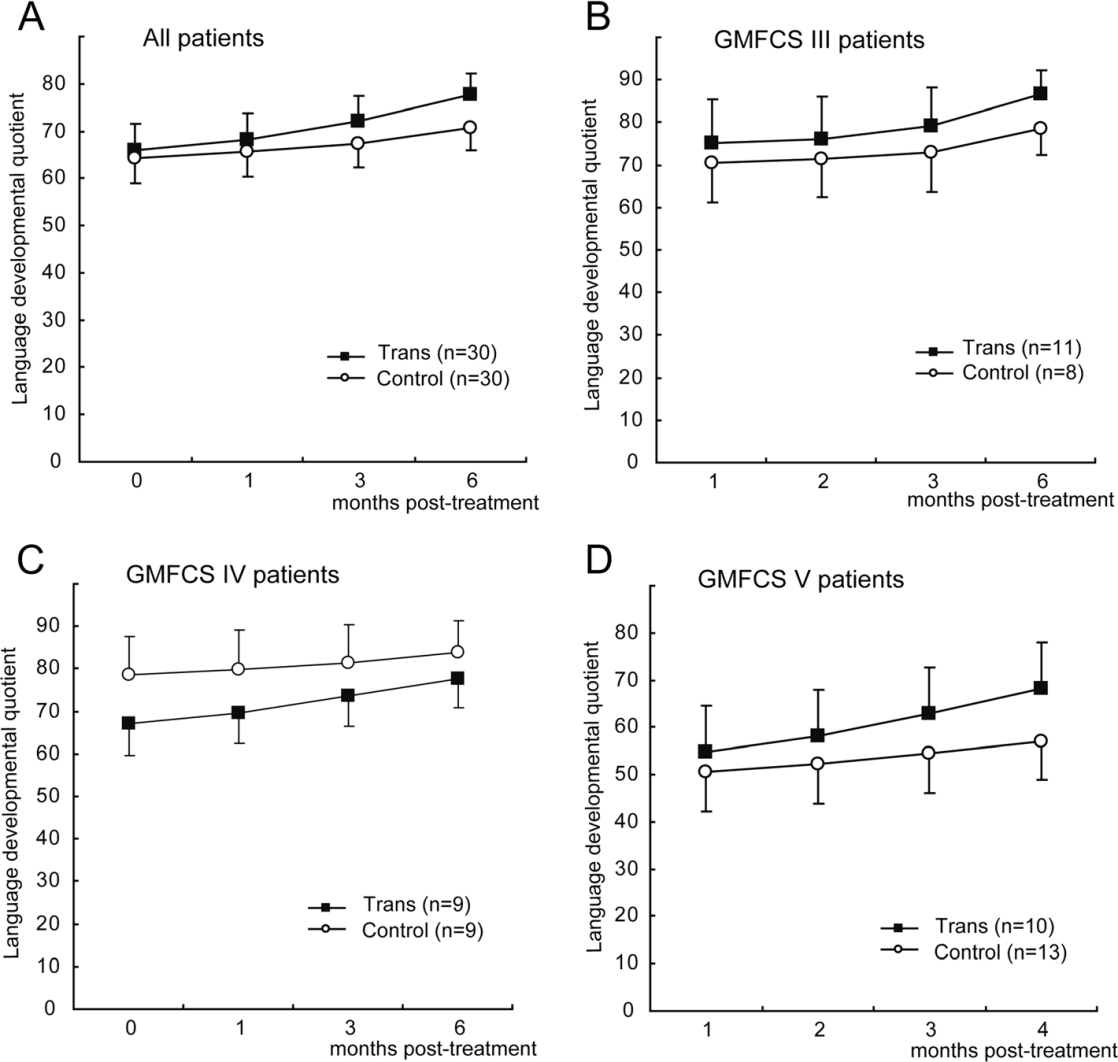
**A comparison of the kinetic language developmental quotients of the patients in the two groups. (A)** A comparison of the mean language developmental quotients before (0 months) and after treatment for the patients in the transplantation (Trans) and control groups. **(B-D)** A comparison of the mean language developmental quotients for the patients with GMFCS levels III **(B)**, IV**(C)** and V **(D)** in the transplantation (Trans) and control groups. No statistically significant differences were detected between the groups using a two-tailed Student’s *t*-test.

The language abilities of the patients were observed before treatment and at 1, 3, and 6 months post-treatment. The means of the language developmental quotients of the patients in the two groups gradually increased during the 6 months post-treatment (Figure 5). However, no significant differences in the language developmental quotients were observed at months 1, 3, and 6 post-treatment when compared with the baseline quotients in both groups (P>0.05 for all). In addition, we did not find any significant differences in the language developmental quotients at months 1, 3, and 6 post-treatment between the two groups (*P=*0.751, 0.522 and 0.304, respectively). This finding suggests that the recovery of language functions was not accelerated in the transplantation group.

### Adverse reactions in the transplantation group

No new neurological deficits were immediately identified after NSC treatment. All of the patients in this study were observed for more than 6 months after treatment. None of the 60 patients experienced fever, headache, allodynia, vomiting, or other serious adverse events that were related to the experimental procedure. Follow-up MRI evaluations did not show any significant anatomical or structural changes that could indicate adverse events. There was no evidence of any new ischemic, haemorrhagic, or neoplastic lesions. However, an increase in the frequency of crying was reported in one patient but resolved spontaneously 2 days after the treatment without any intervention. Other possible adverse effects or complications in the control and transplantation groups were evaluated, but no differences were observed in the frequency of new-onset morbidities between the groups.

## Discussion

Improvements in perinatal emergency medicine have decreased the neonatal mortality rate, however, the incidence of hypoxic ischemic encephalopathy and premature cerebral palsy have increased over time [1]. Conventional rehabilitation treatment for cerebral palsy cannot improve the motor function of patients with moderate-to-severe chronic cerebral palsy [5,6]. In this study, we did not find any significant improvement in the motor functions of the patients in the control group. However, in the transplantation group, our data indicated that the GMFM scores post-treatment were significantly higher after 3 months compared with the baselines scores, which suggests that the motor recovery effects were accelerated in the transplantation group. In addition, we found that patients in the transplantation group with GMFCS levels IV and V had a better recovery of motor functions. These results provide strong clinical support for MSC-derived NSC-like cells transplantation for the treatment of cerebral palsy, especially for moderate-to-severe chronic cerebral palsy.

The mechanism by which NSCs contribute to motor function recovery remains controversial. Experiments have demonstrated that transplanted NSCs exhibit strong plasticity, can easily integrate with host cells and can establish a stable synaptic connection and become functional substituting nerve cells [26-28]. Additionally, NSCs may produce neurotrophic factors that facilitate the recovery of impaired tissues in the diseased brain region [29-32]. Consistent with these findings, we found that autologous NSC-like cells derived from MSCs could differentiate into neuronal and glial lineages in vitro. Our study was not designed to address the mechanism of NSC functions in vivo, and future experiments are necessary to define the exact mechanism of therapeutic cerebral repair by NSCs.

The safety of the NSC-based cell therapy is another urgent problem. The risk for chromosomal aberrations, neoplastic transformation, increased telomerase activity, or both has been reported for human MSCs following several passages in culture in experimental settings [33]. We used early cultured MSCs (3–4 passage) that displayed normal karyotypes and telomerase activity to induce NSC-like cells. Before each treatment, we confirmed the safety of induced NSC-like cells by telomerase activity evaluation, karyotype analysis and microbiological detection. To our knowledge, there are no clinical data that support the development of neoplasms that are directly related to an autologous MSC inoculum [34,35]. In this study, we evaluated the safety of MSCs and NSC-like cells in nude mice, and no tumour formation or other complications were found in a long-term (more than 3 months) study. Moreover, allodynia was reported to be a risk of intraspinal neural stem cell transplantation [30]. In this study, we did not find any cases of allodynia or other adverse events during the 6-month follow-up. Therefore, we confirmed that the cells in our study were safe for the patients, however, more long-term follow-up studies may be necessary to further confirm the safety of NSC-like cells.

In this study, we did not find any evidence of accelerated recovery of language function in the transplantation group. Language disorders in children with cerebral palsy are categorized as asophia, anarthria and language developmental delays [36], which are caused by a motor disturbance of the speech organs due to brain injury. Language recovery is affected by multiple factors [37]. The time period from 7–24 months after birth is important for establishing brain language signal pathways. In this study, all of the patients missed this key phase of language training. Therefore, stem cells combined with language training at an earlier stage may improve the language ability of these patients.

The main limitations of this study include the small sample size and the lack of a randomised, double-blinded, placebo-controlled design. However, we employed an observer-blinded, controlled design to minimize measurement bias. As shown in Figure 3, the baseline characteristics of the patients from the two groups are well matched, and no significant differences were found between the two groups in the comparison of the patient ages, LDQs and GMFM scores at the study onset. This study is the first reported controlled clinical trial of NSC-like cells therapy for chronic cerebral palsy, and our study provides strong clinical evidence that supports stem cell transplantation for the treatment of motor deficits related to cerebral palsy. Further randomised clinical trials are necessary to establish the efficacy of this procedure.

## Conclusion

Our data indicates that the transplantation of MSC-derived NSC-like cells is safe and effective for the treatment of chronic cerebral palsy. Motor function, but not the language quotient, indicated optimal improvement 3 months after transplantation.

## Competing interests

The authors declare that they have no competing interests.

## Authors’ contributions

HQL conceived of the study, participated in its design, and drafted and revised the manuscript. GJC and YLW participated in the design of the study, collected and analysed the data, and drafted the manuscript. ZYX participated in the design of the study, analysed the data, drafted and revised the manuscript and performed the immunoassays. FF and RMX collected and analysed the data. YW drafted and revised the manuscript and performed the statistical analysis. XLH and LXF participated in the coordination of the study and helped draft the manuscript. All of the authors read and approved the final manuscript.

## Supplementary Material

Additional file 1 Supplementary materials. Click here for file

Additional file 2 The motor ability of patient No. 4 in the transplantation group before and after treatment. Click here for file

Additional file 3 The motor ability of patient No. 5 in the transplantation group before and after treatment. Click here for file
