# Supplementary material for: Neural stem cell-like cells derived from autologous bone mesenchymal stem cells for the treatment of patients with cerebral palsy
Source: J Transl Med. 2013 Jan 26;11:21. doi: 10.1186/1479-5876-11-21 (PMC3563497; doi:10.1186/1479-5876-11-21)
Supplement: Additional file 1 — Supplementary materials. [file 1479-5876-11-21-S1.doc]

**Supplementary Methods**

## Preparation of autologous MSCs

The methods that were used for the bone marrow aspiration, isolation of MSCs, cell culture, cell preparation, and i.v. infusions were performed according to previously published methods with several modifications [[^14^](#_ENREF_14)]**.** The patients fasted for 4-6 hours and went without water 1 hour before the collection of autologous bone marrow specimens. The patients were injected with 5 mg/kg phenobarbital, 0.01 mg/kg atropine, and 4 mg/kg ketamine in the aseptic collecting room 30 minutes before specimen collection, and 8-25 mL of bone marrow (0.6-1.0 mL/kg of body weight) was collected from the right posterior superior iliac spine according to conventional bone marrow aspiration procedures. A total of 100 U/mL heparin was added to the collecting tube as an anticoagulant. The specimens were sent to the laboratory immediately after collection.

## Separation and culture of MSCs

Bone marrow was placed in a 15 mL centrifuge tube followed by centrifugation at 1,000 R/minute for 7 minutes. The white membrane component in the middle of the tube, which contained the smallest number of red cells, was transferred into another tube with a tubule, washed twice with PBS and centrifuged at 1000 R/minute for 7 minutes. The supernatant was removed, and the cells were re-suspended in culture medium and placed in a Petri dish. The cells were incubated at a saturated humidity, 5% CO_2_ and 37°C. Half of the medium was changed after 48 hours, and all of the medium was replaced on day 4 to remove the cells in the suspension. Subsequently, the medium was changed based on colour changes. After reaching 60%-80% confluency, the primary cells were infinitely dilated and placed in a 96-well plate for cloning. The cells with a strong positive signal for the multipotential marker Oct4 and Nanog were screened for further cloning and culturing (Figure S1A).

## Identification and usage standard of MSCs

When the cell population reached 10^8^, the cells were washed twice with PBS. A 0.05% Trypsin-EDTA solution was used to digest the cells, and culture medium was added to stop the digestion and prepare 1-3×10^6^ cell suspensions. To determine the phenotype of the cultured MSCs, the collected cells were assayed using flow cytometry. The results indicated that CD34 and CD45 were negative, and the positive rates of CD29 and CD44 were above 95% (Figure S1B). Then, the cells were used for the induction of neural stem cells or transplantation. Before treatment, the safety of the cells was confirmed by assessing telomerase activity, and the supernatant tested negative for the Epstein-Barr virus, cytomegalovirus, hepatitis B virus, mycoplasma, bacteria, and fungi. The MSCs were continuously cultured without cryopreservation and were thawed before transplantation.

****Supplementary** Table 1 –Examples of the Gross Motor Function Measure questionnaire.**

| **GMFM scores for CP patients** | | | | | | | |  | | | |  |
| --- | --- | --- | --- | --- | --- | --- | --- | --- | --- | --- | --- | --- |
| **name: Case 10 age: 33 month sex: female GMFCS Level : III Evaluator’s Name: RenMei-Xu** | | | | | | | | | | | |  |
| Program | | Rating | | | | | | **socre** | | | |  |
| **A: LYING & ROLLING** | | | | | | | |  | | | |  |
| 1 | SUP, HEAD IN MIDLINE: TURNS HEAD WITH EXTREMITIES SYMMETRICAL | 0 | 1 | | 2 | 3 | | | **3** | | |  |
| 2 | SUP: BRINGS HANDS TO MIDLINE, FINGERS ONE WITH THE OTHER | 0 | 1 | | 2 | 3 | | | **3** | | |  |
| 3 | SUP: LIFTS HEAD 45° | 0 | 1 | | 2 | 3 | | | **3** | | |  |
| 4 | SUP: FLEXES R HIP AND KNEE THROUGH FULL RANGE | 0 | 1 | | 2 | 3 | | | **3** | | |  |
| 5 | SUP: FLEXES L HIP AND KNEE THROUGH FULL RANGE | 0 | 1 | | 2 | 3 | | | **3** | | |  |
| 6 | SUP: REACHES OUT WITH R ARM, HAND CROSSES MIDLINE TOWARD TOY | 0 | 1 | | 2 | 3 | | | **3** | | |  |
| 7 | SUP: REACHES OUT WITH L ARM, HAND CROSSES MIDLINE TOWARD TOY | 0 | 1 | | 2 | 3 | | | **3** | | |  |
| 8 | SUP: ROLLS TO PR OVER R SIDE | 0 | 1 | | 2 | 3 | | | **3** | | |  |
| 9 | SUP: ROLLS TO PR OVER L SIDE | 0 | 1 | | 2 | 3 | | | **3** | | |  |
| 10 | PR: LIFTS HEAD UPRIGHT | 0 | 1 | | 2 | 3 | | | **3** | | |  |
| 11 | PR ON FOREARMS: LIFTS HEAD UPRIGHT, ELBOWS EXT, CHEST RAISED | 0 | 1 | | 2 | 3 | | | **3** | | |  |
| 12 | PR ON FOREARMS: WEIGHT ON R FOREARM, FULLY EXTENDS OPPOSITE ARM FORWARD | 0 | 1 | | 2 | 3 | | | **3** | | |  |
| 13 | PR ON FOREARMS: WEIGHT ON L FOREARM, FULLY EXTENDS OPPOSITE ARM FORWARD | 0 | 1 | | 2 | 3 | | | **3** | | |  |
| 14 | PR: ROLLS TO SUP OVER R SIDE | 0 | 1 | | 2 | 3 | | | **3** | | |  |
| 15 | PR: ROLLS TO SUP OVER L SIDE | 0 | 1 | | 2 | 3 | | | **3** | | |  |
| 16 | PR: PIVOTS TO R 90° USING EXTREMITIES | 0 | 1 | | 2 | 3 | | | **3** | | |  |
| 17 | PR: PIVOTS TO L 90° USING EXTREMITIES | 0 | 1 | | 2 | 3 | | | **3** | | |  |
| **B: SITTING** | | | | | | | | |  | | |  |
| 18 | SUP, HANDS GRASPED BY EXAMINER: PULLS SELF TO SITTING WITH HEAD CONTROL | 0 | 1 | | 2 | 3 | | | **3** | | |  |
| 19 | SUP: ROLLS TO R SIDE, ATTAINS SITTING | 0 | 1 | | 2 | 3 | | | **3** | | |  |
| 20 | SUP: ROLLS TO L SIDE, ATTAINS SITTING | 0 | 1 | | 2 | 3 | | | **3** | | |  |
| 21 | SIT ON MAT, SUPPORTED AT THORAX BY THERAPIST: LIFTS HEAD UPRIGHT, MAINTAINS 3 SECONDS | 0 | 1 | | 2 | 3 | | | **3** | | |  |
| 22 | SIT ON MAT, SUPPORTED AT THORAX BY THERAPIST: LIFTS HEAD MIDLINE, MAINTAINS 10 SECONDS | 0 | 1 | | 2 | 3 | | | **3** | | |  |
| 23 | SIT ON MAT, ARM(S) PROPPING: MAINTAINS, 5 SECONDS | 0 | 1 | | 2 | 3 | | | **3** | | |  |
| 24 | SIT ON MAT: MAINTAINS, ARMS FREE, 3 SECONDS | 0 | 1 | | 2 | 3 | | | **3** | | |  |
| 25 | SIT ON MAT WITH SMALL TOY IN FRONT: LEANS FORWARD, TOUCHES TOY, RE-ERECTS WITHOUT ARM PROPPING | 0 | 1 | | 2 | 3 | | | **1** | | |  |
| 26 | SIT ON MAT: TOUCHES TOY PLACED 45° BEHIND CHILD’S R SIDE, RETURNS TO START | 0 | 1 | | 2 | 3 | | | **0** | | |  |
| 27 | SIT ON MAT: TOUCHES TOY PLACED 45° BEHIND CHILD’S L SIDE, RETURNS TO START | 0 | 1 | | 2 | 3 | | | **0** | | |  |
| 28 | R SIDE SIT: MAINTAINS, ARMS FREE, 5 SECONDS | 0 | 1 | | 2 | 3 | | | **1** | | |  |
| 29 | L SIDE SIT: MAINTAINS, ARMS FREE, 5 SECONDS | 0 | 1 | | 2 | 3 | | | **3** | | |  |
| 30 | SIT ON MAT: LOWERS TO PR WITH CONTROL | 0 | 1 | | 2 | 3 | | | **0** | | |  |
| 31 | SIT ON MAT WITH FEET IN FRONT: ATTAINS 4 POINT OVER R SIDE | 0 | 1 | | 2 | 3 | | | **0** | | |  |
| 32 | SIT ON MAT WITH FEET IN FRONT: ATTAINS 4 POINT OVER L SIDE | 0 | 1 | | 2 | 3 | | | **0** | | |  |
| 33 | SIT ON MAT: PIVOTS 90°, WITHOUT ARMS ASSISTING | 0 | 1 | | 2 | 3 | | | **0** | | |  |
| 34 | SIT ON BENCH: MAINTAINS, ARMS AND FEET FREE, 10 SECONDS | 0 | 1 | | 2 | 3 | | | **1** | | |  |
| 35 | STD: ATTAINS SIT ON SMALL BENCH | 0 | 1 | | 2 | 3 | | | **1** | | |  |
| 36 | ON THE FLOOR: ATTAINS SIT ON SMALL BENCH | 0 | 1 | | 2 | 3 | | | **0** | | |  |
| 37 | ON THE FLOOR: ATTAINS SIT ON LARGE BENCH | 0 | 1 | | 2 | 3 | | | **0** | | |  |
| **C: CRAWLING & KNEELING** | | | | | | | | | | |  | |
| 38 | PR: CREEPS FORWARD 2m | 0 | 1 | | 2 | 3 | | | **1** | | |  |
| 39 | 4 POINT: MAINTAINS, WEIGHT ON HANDS AND KNEES, 10 SECONDS | 0 | 1 | | 2 | 3 | | | **1** | | |  |
| 40 | 4 POINT: ATTAINS SIT ARMS FREE | 0 | 1 | | 2 | 3 | | | **0** | | |  |
| 41 | PR: ATTAINS 4 POINT, WEIGHT ON HANDS AND KNEES | 0 | 1 | | 2 | 3 | | | **0** | | |  |
| 42 | 4 POINT: REACHES FORWARD WITH R ARM, HAND ABOVE SHOULDER LEVEL | 0 | 1 | | 2 | 3 | | | **0** | | |  |
| 43 | 4 POINT: REACHES FORWARD WITH L ARM, HAND ABOVE SHOULDER LEVEL | 0 | 1 | | 2 | 3 | | | **0** | | |  |
| 44 | 4 POINT: CRAWLS OR HITCHES FORWARD 2m | 0 | 1 | | 2 | 3 | | | **0** | | |  |
| 45 | 4 POINT: CRAWLS RECIPROCALLY FORWARD 2m | 0 | 1 | | 2 | 3 | | | **0** | | |  |
| 46 | 4 POINT: CRAWLS UP 4 STEPS ON HANDS AND KNEES/FEET | 0 | 1 | | 2 | 3 | | | **0** | | |  |
| 47 | 4 POINT: CRAWLS BACKWARDS DOWN 4 STEPS ON HANDS AND KNEES/FEET | 0 | 1 | | 2 | 3 | | | **0** | | |  |
| 48 | SIT ON MAT: ATTAINS HIGH KN USING ARMS, MAINTAINS, ARMS FREE, 10 SECONDS | 0 | 1 | | 2 | 3 | | | **0** | | |  |
| 49 | HIGH KN: ATTAINS HALF KN ON R KNEE USING ARMS, MAINTAINS, ARMS FREE, 10 SECONDS | 0 | 1 | | 2 | 3 | | | **0** | | |  |
| 50 | HIGH KN: ATTAINS HALF KN ON L KNEE USING ARMS, MAINTAINS, ARMS FREE, 10 SECONDS | 0 | 1 | | 2 | 3 | | | **0** | | |  |
| 51 | HIGH KN: KN WALKS FORWARD 10 STEPS, ARMS FREE | 0 | 1 | | 2 | 3 | | | **0** | | |  |
| **D: STANDING** | | | | | | | | |  | | |  |
| 52 | ON THE FLOOR: PULLS TO STD AT LARGE BENCH | 0 | 1 | | 2 | 3 | | | **3** | | |  |
| 53 | STD: MAINTAINS, ARMS FREE, 3 SECONDS | 0 | 1 | | 2 | 3 | | | **3** | | |  |
| 54 | STD: HOLDING ON TO LARGE BENCH WITH ONE HAND, LIFTS R FOOT, 3 SECONDS | 0 | 1 | | 2 | 3 | | | **1** | | |  |
| 55 | STD: HOLDING ON TO LARGE BENCH WITH ONE HAND, LIFTS L FOOT, 3 SECONDS | 0 | 1 | | 2 | 3 | | | **1** | | |  |
| 56 | STD: MAINTAINS, ARMS FREE, 20 SECONDS | 0 | 1 | | 2 | 3 | | | **3** | | |  |
| 57 | STD: LIFTS L FOOT, ARMS FREE, 10 SECONDS | 0 | 1 | | 2 | 3 | | | **1** | | |  |
| 58 | STD: LIFTS R FOOT, ARMS FREE, 10 SECONDS | 0 | 1 | | 2 | 3 | | | **1** | | |  |
| 59 | SIT ON SMALL BENCH: ATTAINS STD WITHOUT USING ARMS | 0 | 1 | | 2 | 3 | | | **1** | | |  |
| 60 | HIGH KN: ATTAINS STD THROUGH HALF KN ON R KNEE, WITHOUT USING ARMS | 0 | 1 | | 2 | 3 | | | **1** | | |  |
| 61 | HIGH KN: ATTAINS STD THROUGH HALF KN ON L KNEE, WITHOUT USING ARMS | 0 | 1 | | 2 | 3 | | | **1** | | |  |
| 62 | STD: LOWERS TO SIT ON FLOOR WITH CONTROL, ARMS FREE | 0 | 1 | | 2 | 3 | | | **1** | | |  |
| 63 | STD: ATTAINS SQUAT, ARMS FREE | 0 | 1 | | 2 | 3 | | | **1** | | |  |
| 64 | STD: PICKS UP OBJECT FROM FLOOR, ARMS FREE, RETURNS TO STAND | 0 | 1 | | 2 | 3 | | | **1** | | |  |
| **E: WALKING, RUNNING & JUMPING** | | | | | | | | |  | | |  |
| 65 | STD, 2 HANDS ON LARGE BENCH: CRUISES 5 STEPS TO R | 0 | 1 | | 2 | 3 | | | **3** | | |  |
| 66 | STD, 2 HANDS ON LARGE BENCH: CRUISES 5 STEPS TO L | 0 | 1 | | 2 | 3 | | | **1** | | |  |
| 67 | STD, 2 HANDS HELD: WALKS FORWARD 10 STEPS | 0 | 1 | | 2 | 3 | | | **1** | | |  |
| 68 | STD, 1 HAND HELD: WALKS FORWARD 10 STEPS | 0 | 1 | | 2 | 3 | | | **0** | | |  |
| 69 | STD: WALKS FORWARD 10 STEPS | 0 | 1 | | 2 | 3 | | | **0** | | |  |
| 70 | STD: WALKS FORWARD 10 STEPS, STOPS, TURNS 180°, RETURNS | 0 | 1 | | 2 | 3 | | | **0** | | |  |
| 71 | STD: WALKS BACKWARD 10 STEPS | 0 | 1 | | 2 | 3 | | | **0** | | |  |
| 72 | STD: WALKS FORWARD 10 STEPS, CARRYING A LARGE OBJECT WITH 2 HANDS | 0 | 1 | | 2 | 3 | | | **0** | | |  |
| 73 | STD: WALKS FORWARD 10 CONSECUTIVE STEPS BETWEEN PARALLEL LINES 20cm (8") APART | 0 | 1 | | 2 | 3 | | | **0** | | |  |
| 74 | STD: WALKS FORWARD 10 CONSECUTIVE STEPS ON A STRAIGHT LINE 2cm (3/4") WIDE | 0 | 1 | | 2 | 3 | | | **0** | | |  |
| 75 | STD: STEPS OVER STICK AT KNEE LEVEL, R FOOT LEADING | 0 | 1 | | 2 | 3 | | | **0** | | |  |
| 76 | STD: STEPS OVER STICK AT KNEE LEVEL, L FOOT LEADING | 0 | 1 | | 2 | 3 | | | **0** | | |  |
| 77 | STD: RUNS 5m , STOPS & RETURNS | 0 | 1 | | 2 | 3 | | | **0** | | |  |
| 78 | STD: KICKS BALL WITH R FOOT | 0 | 1 | | 2 | 3 | | | **0** | | |  |
| 79 | STD: KICKS BALL WITH L FOOT | 0 | 1 | | 2 | 3 | | | **0** | | |  |
| 80 | STD: JUMPS FORWARD5 cm , BOTH FEET SIMULTANEOUSLY | 0 | 1 | | 2 | 3 | | | **0** | | |  |
| 81 | STD: JUMPS FORWARD 30 cm (12"), BOTH FEET SIMULTANEOUSLY | 0 | 1 | | 2 | 3 | | | **0** | | |  |
| 82 | STD ON R FOOT: HOPS ON R FOOT 10 TIMES WITHIN A 60cm (24") CIRCLE | 0 | 1 | | 2 | 3 | | | **0** | | |  |
| 83 | STD ON L FOOT: HOPS ON L FOOT 10 TIMES WITHIN A 60cm (24") CIRCLE | 0 | 1 | | 2 | 3 | | | **0** | | |  |
| 84 | STD, HOLDING 1 RAIL: WALKS UP 4 STEPS, HOLDING 1 RAIL, ALTERNATING FEET | 0 | 1 | | 2 | 3 | | | **0** | | |  |
| 85 | STD, HOLDING 1 RAIL: WALKS DOWN 4 STEPS, HOLDING 1 RAIL, ALTERNATING FEET | 0 | 1 | | 2 | 3 | | | **0** | | |  |
| 86 | STD: WALKS UP 4 STEPS, ALTERNATING FEET | 0 | 1 | | 2 | 3 | | | **0** | | |  |
| 87 | STD: WALKS DOWN 4 STEPS, ALTERNATING FEET | 0 | 1 | | 2 | 3 | | | **0** | | |  |
| 88 | STD ON 15cm (6") STEP: JUMPS OFF, BOTH FEET SIMULTANEOUSLY | 0 | 1 | | 2 | 3 | | | **0** | | |  |
|  | | | | | | | | **105** | | | |  |
|  |  |  | |  | | |  | | |  | |  |
|  |  | date:2010-7-2 | | | | |  | | |  | |  |

## **Supplementary** Figure

##
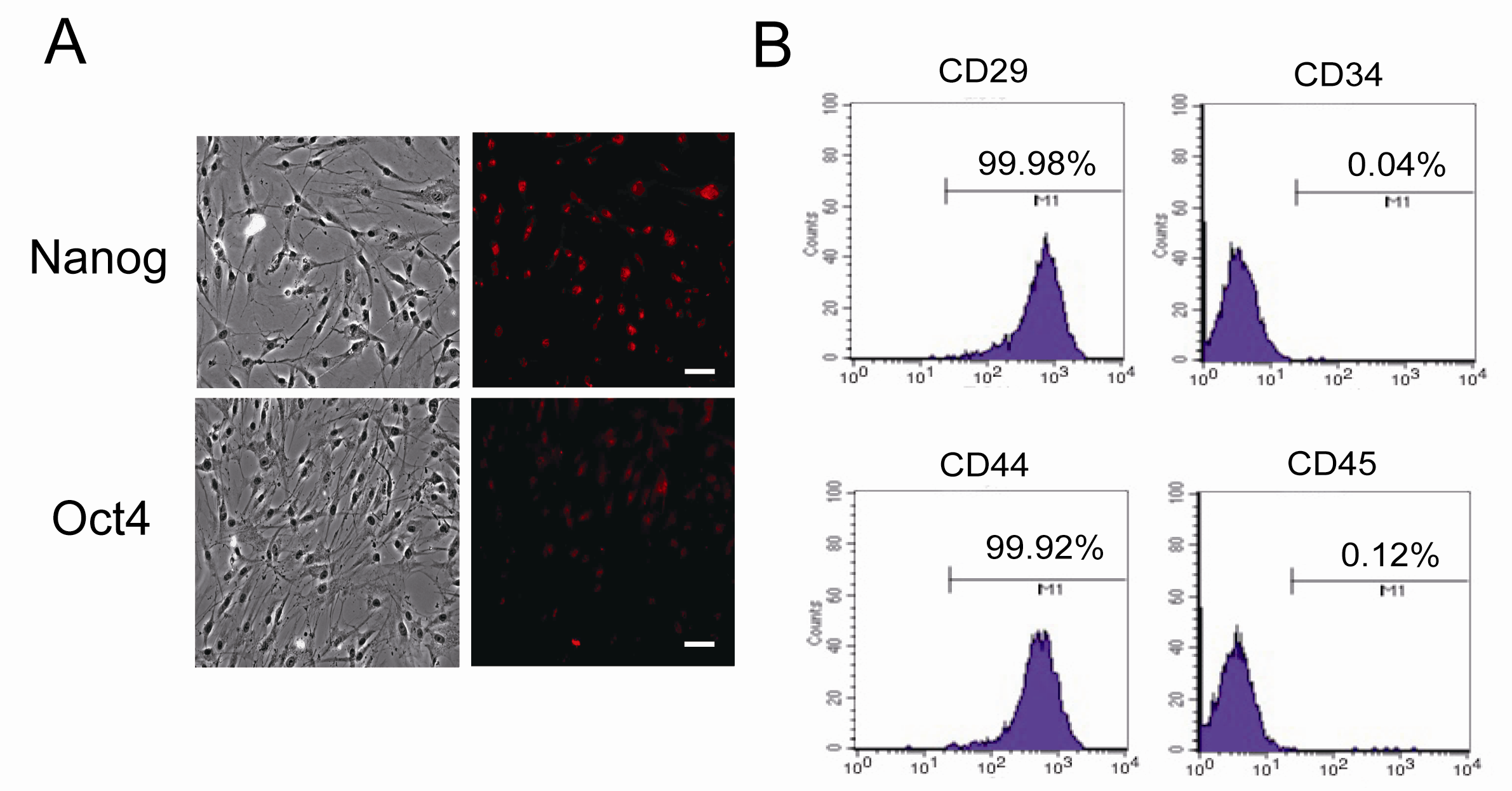


## **Supplementary** Figure 1 - Characterisation of bone marrow-derived MSCs

MSCs were fixed and stained using antibodies against Nanog and Oct4 and were visualized with a PE-labelled secondary antibody. The scale bar is 10 μm (A). MSCs were harvested and labeled with antibodies against CD29, CD34, CD44, CD45, or control IgGs and analysed using FACS. The rate of positive cells is shown. The tests were repeated at least three times (B).
